# Occupational lung disease: What the general physician needs to know

**DOI:** 10.1016/j.clinme.2025.100305

**Published:** 2025-04-02

**Authors:** Patrick Howlett, Joanna Szram, Johanna Feary

**Affiliations:** a National Heart & Lung Institute, Imperial College London, Guy Scadding Building, Cale Street, London, SW3 6LY, United Kingdom; b Occupational Lung Disease Department, Royal Brompton Hospital, Sydney St, London SW3 6NP, United Kingdom

**Keywords:** Occupation, Lung

## Abstract

Occupational exposures are a common and preventable cause of lung disease. About one in six cases of chronic obstructive pulmonary disease (COPD) and asthma worldwide are related to work.

Early recognition of occupational lung disease improves outcomes. Doctors should ask about work history in patients with respiratory symptoms. This educational review article briefly outlines key clinical features, relevant to the general physician, of common occupational lung diseases seen in the UK. These conditions include work-related asthma, pneumoconioses, hypersensitivity pneumonitis and COPD.

Referral to a specialist is recommended when an occupational cause is suspected. Most occupational lung diseases are preventable with adequate workplace safety measures and early medical attention.

## Highlights

• Occupational exposures are an underestimated cause of respiratory disease, including one in six cases of chronic obstructive pulmonary disease and asthma.
• A concise yet careful history can help determine whether there is an occupational cause for a patient's presentation.
• Refer to a specialist occupational lung disease centre as soon as an occupational cause is suspected.
• There has been a recent increase in the number of silicosis cases in the UK; vigilance is needed for cases.
• Occupational lung diseases are largely preventable with appropriate monitoring and safeguards.

## Introduction

Occupational exposures are a common, often overlooked and – in most cases – entirely preventable cause of lung disease. For both chronic obstructive pulmonary disease (COPD) and asthma, approximately one in six cases worldwide are work-related, resulting in a loss of nine and two million disability-adjusted life years, respectively.^1^ In the UK, there are an estimated 12,000 deaths from occupational exposures each year, around 60% of which are from cancer.^2^

## Detecting work-related respiratory disease

Early specialist input can lead to better outcomes for patients with occupational lung diseases.^3^ This requires a suspicion that respiratory symptoms, clinical findings or a diagnosis may be work-related at presentation to an acute medical unit, general practice or respiratory clinic.

Occupational lung diseases are frequently characterised by their latency relating to work factors – causative or exacerbating. This ranges from immediate onset (no latency), such as acute inhalation injury from exposure to high-dose toxic airborne agents, through to the 40-year latency of mesothelioma following asbestos exposure. Conventionally, diseases are categorised into those of short latency (occupational asthma, hypersensitivity pneumonitis) and long latency (asbestos-related lung diseases, silicosis, other pneumoconioses, lung cancers and COPD) usually occurring at least 10 years after first exposure.

While a knowledge of the common causative agents of occupational lung disease is helpful, there are many hundreds of known respiratory hazards in the workplace, with more identified and described each year. Therefore, guidelines recommend enquiry about current or past job roles related to the time course of the disease in question, and an understanding of whether reported respiratory symptoms are worse when at work, and improve away from work. A resulting suspicion of a work relationship should prompt early referral to an occupational lung disease specialist.

Following diagnosis of an occupational lung disease, a careful discussion should be held with the patient about the risks and benefits of continuing work (if they remain exposed to the causal agent) to achieve the best balance between long-term health and employment outcomes. Simply advising someone to stop work may be neither realistic nor in their best interests, depending on their personal circumstances.

## Work-related asthma

One in six cases of adult asthma is attributable to occupational exposures. Clinicians should ask about the relationship between work and symptoms in all patients with asthma and particularly in cases of adult-onset or relapsed childhood asthma. Three categories of work-related asthma are summarised below, with further details – including screening questions and referral guidance – in the BTS Occupational Asthma Clinical Statement.^3^

**‘Work-aggravated asthma’ (WAA)** is pre-existing asthma triggered by workplace exposures (eg irritants like chemicals and dust, physical exertion, cold and shift changes. It is more common in those with poorly controlled asthma and is usually managed with improved workplace exposure control and conventional asthma treatment.

**Occupational asthma (OA)** should be distinguished from WAA and is induced by exposure to airborne proteins (high molecular mass) or reactive chemicals (low molecular mass); there are around 400 known workplace sensitisers (Table 1). It can develop in workers with or without pre-existing asthma. It begins with a symptom-free latent period, which varies from a few weeks to years – although the risk of OA is highest in the first year of exposure – followed by onset of work-related respiratory symptoms that worsen during or after work and improve on days away from work. Occupational rhinitis frequently coexists in OA due to proteins.

**Table 1:** Common causes of occupational asthma.

Exposure	Common occupations
**High molecular mass**	
Animal proteins	Farming, poultry work, veterinary medicine, laboratory work
Flour and cereal proteins⁎	Baking, food processing
Latex	Healthcare, textile work
**Low molecular mass**	
Diisocyanates⁎	Spray painting, foam manufacture, polyurethane work
Acid anhydrides	Paint and plastic manufacture
Glues and resins	Plastic manufacture, material engineering, dental technology, nail salon work, construction
Drugs (penicillins, opiates)	Pharmaceutical manufacture
Wood dust (eg western red cedar)	Woodwork
Dyes and bleaches	Hairdressing, textile work
Precious metals (eg platinum salts)	Precious metal refining

⁎ Two most common causal agents in OA in the UK.^4^

Investigation of OA is complex and should be undertaken by clinicians with appropriate specialist expertise. Immunological tests (serum-specific IgE or skin-prick tests) can confirm sensitisation to putative causal agents. These are generally available for all protein allergens, either commercially or as bespoke assays available in specialist centres. Immunological tests are also available for a limited number of chemical agents (eg isocyanates and acid anhydrides). Detailed serial peak flow measurement, with measurements recorded at least four times daily over three consecutive weeks and including days at and away from work, is the most accessible diagnostic tool for OA. Peak flow records should be interpreted in the context of the clinical history (including immunology) and using specialist software or by experienced clinicians. Specific inhalation challenge testing at specialist centres is indicated for selected cases.

Management of OA includes advice about removal (or reduction) of exposure, prescription of standard asthma medications, treatment of related comorbidities, advice about benefits and compensation, and often involves liaison with the employer/occupational health.^3^ Distinction from WAA and accurate diagnosis is crucial to prevent unnecessary career disruption. OA is curable if identified early, but is frequently diagnosed late, by which time cases have progressed to severe and, in some cases, irreversible disease. Prognosis is largely determined by asthma severity at the time of diagnosis, and whether workers have ongoing exposure to the causal agent.^3^ Around one in three people with OA are unemployed 3–5 years after diagnosis.

**Irritant-induced asthma (IIA)**, formerly known as reactive airways dysfunction syndrome (RADS), results from inflammation and repair rather than an immune response. It typically follows acute high-dose irritant exposure with development of symptoms within 24 h. Diagnosis should include objective tests of asthma, eg bronchial hyper-responsiveness, and efforts made to exclude other conditions such as breathing pattern dysfunction and post-traumatic stress disorder.^3^ Treatment is with standard asthma therapy and prognosis is varied but symptoms and bronchial hyper-responsiveness may last up to decades after initial exposure.^5^

## Pneumoconioses

Some inorganic dusts inhaled at work are biologically inert while others, such as asbestos, silica and coal mine dust, cause fibrosis and lung dysfunction. These diseases typically have a long latency period and can progress even after exposure has ended. Common types including silicosis, coal workers’ pneumoconiosis and asbestosis.

**Fig. 1:**
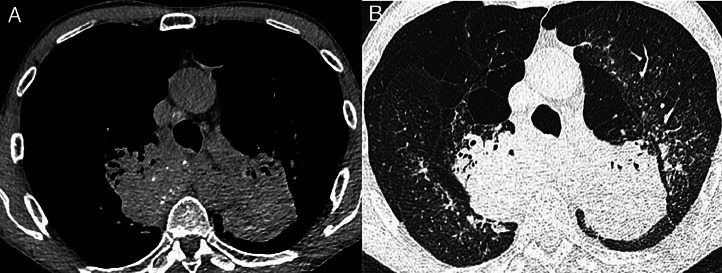
(A) Axial CT section (soft-tissue windows) showing complicated silicosis with progressive massive fibrosis. There are bilateral perihilar conglomerate masses with punctate calcification. Mediastinal calcified lymph nodes are also demonstrated. (B) Lung window settings at the same level show coexisting nodularity and emphysema.^7^

**Silicosis** is caused by inhaling respirable crystalline silica and primarily occurs in industries such as stone work, mining, ceramics, foundry work and sandblasting.^6^ Clinical and radiological similarities with tuberculosis and sarcoidosis mean that silicosis cases are easily overlooked unless a careful occupational history is taken. Presentation ranges from asymptomatic simple nodular silicosis to more ‘complicated’ or progressive massive fibrosis (PMF) disease, associated with respiratory impairment. ‘Accelerated’ disease occurs within 10 years of exposure, while acute silicosis is phenotypically distinct (with radiological features of alveolar proteinosis), occurs following high-intensity exposure and is often rapidly progressive (Fig. 1). Cases of artificial stone silicosis in workers fabricating kitchen worktops have surged since 2023 in the UK,^7^ reflecting a global pattern over the last decade.^8^ These cases typically involve young individuals with accelerated disease, sometimes with autoimmune features; morbidity and mortality are high. Silica exposure is also associated with tuberculosis, COPD and lung cancer.

Like silicosis, simple **coal workers’ pneumoconiosis** presents with upper lobe nodular pattern on imaging and few symptoms, but may progress to PMF.

**Asbestosis** resulting from asbestos exposure presents radiographically as usual interstitial pneumonia pattern, hence it is often indistinguishable from idiopathic pulmonary fibrosis. A careful occupational history is key to identifying relevant exposure with highest exposures occurring in pipe fitters and plumbers, boat builders/repairers, carpenters and construction workers and others. There is a dose response between asbestos exposure and risk of disease, with a latency of around 20–30 years. Asbestos exposure also causes asymptomatic pleural plaques, diffuse pleural thickening, benign pleural effusions, lung cancer and malignant mesothelioma.^9^ There are currently over 5,000 asbestos-related deaths per annum in the UK.^10^

Management of pneumoconiosis is best coordinated with a specialist occupational lung disease centre; if progressive undifferentiated fibrosis is present, antifibrotics may improve spirometry outcomes.^11^ Complete cessation of exposure is ideal but if the patient is still working, as with other occupational lung diseases, the relative risks and benefits of stopping should be carefully discussed. Advanced cases may be suitable for transplantation.

## Hypersensitivity pneumonitis

The list of known causes of hypersensitivity pneumonitis is extensive; approximately one in three cases are considered to have an occupational cause.^12^ The most common occupational causes in the UK are contaminated metal working fluid, mouldy hay, and bird feather or dropping proteins.^13^ Cases may occur sporadically or in outbreaks.^14^ Clinical course is variable and a work-related symptom pattern often unclear. Prognosis is improved by identifying and avoiding the causative agent.

## COPD

Occupational causes of COPD include coal dust, cadmium, silica exposure and vapour, gases, dusts and fumes. However, smoking remains the main cause of COPD in most occupational groups. Cases of accelerated declining lung (between 10% and 15% of FEV_1_ in 1 year) may be detected through workplace spirometry surveillance and can prompt identification and modification of causal exposures.^15^

## Other diseases

Other diseases with occupational aetiology include obliterative bronchiolitis, for example related to diacetyl exposure in the manufacture of popcorn,^16^ and byssinosis due to cotton dust exposure.^17^

## Compensation

The UK operates a statutory workers’ compensation scheme through the Industrial Injuries Disablement Benefit (IIDB), covering prescribed occupational diseases. Financial support is based on disability severity.^18^ If there is concern of negligence or a breach of statutory duty, legal proceedings against the employer may be pursued by the patient. Importantly, in almost all cases, proceedings must start within 3 years of diagnosis due to the statute of limitations, highlighting the need for early specialist occupational lung disease input.

## Funding

The authors declare no financial conflicts of interest related to this work. PH is supported by an MRC Clinical Research Training Fellowship (MR/W024861/1).

## CRediT authorship contribution statement

**Patrick Howlett:** Writing – review & editing, Writing – original draft. **Joanna Szram:** Writing – review & editing. **Johanna Feary:** Writing – review & editing, Writing – original draft.

## Declaration of competing interests

The authors declare that they have no known competing financial interests or personal relationships that could have appeared to influence the work reported in this paper.
